# Pan-genomic analysis of *Corynebacterium amycolatum* gives insights into molecular mechanisms underpinning the transition to a pathogenic phenotype

**DOI:** 10.3389/fmicb.2022.1011578

**Published:** 2022-11-16

**Authors:** Hendor N. R. Jesus, Danilo J. P. G. Rocha, Rommel T. J. Ramos, Artur Silva, Bertram Brenig, Aristóteles Góes-Neto, Mateus M. Costa, Siomar C. Soares, Vasco Azevedo, Eric R. G. R. Aguiar, Luiz Martínez-Martínez, Alain Ocampo, Sana Alibi, Alexis Dorta, Luis G. C. Pacheco, Jesus Navas

**Affiliations:** ^1^Multicenter Post-Graduate Program in Biochemistry and Molecular Biology (PMBqBM), Institute of Health Sciences, Federal University of Bahia, Salvador, BA, Brazil; ^2^Post-Graduate Program in Biotechnology, Institute of Health Sciences, Federal University of Bahia, Salvador, BA, Brazil; ^3^Institute of Biological Sciences, Federal University of Para, Belém, PA, Brazil; ^4^Institute of Veterinary Medicine, University of Göttingen, Göttingen, Germany; ^5^Institute of Biological Sciences, Federal University of Minas Gerais, Belo Horizonte, MG, Brazil; ^6^Laboratório de Microbiologia e Imunologia Animal (LAMIA), Universidade Federal Do Vale Do São Francisco, Petrolina, Pernambuco, Brazil; ^7^Department of Immunology, Microbiology and Parasitology, Institute of Biological and Natural Sciences, Federal University of Triângulo Mineiro (UFTM), Uberaba, Minas Gerais, Brazil; ^8^Department of Biological Sciences, State University of Santa Cruz, Ilhéus, BA, Brazil; ^9^Unidad de Gestión Clínica, Hospital Universitario Reina Sofía, Córdoba, Spain; ^10^Departamento de Microbiología, Universidad de Córdoba, Córdoba, Spain; ^11^Instituto Maimónides de Investigación Biomédica de Córdoba (IMIBIC), Córdoba, Spain; ^12^Microbiology Service, University Hospital Marqués de Valdecilla, Santander, Spain; ^13^Instituto de Investigación Valdecilla (IDIVAL), Santander, Spain; ^14^Research Unit Analysis and Process Applied to the Environment, Rejiche, Tunisia; ^15^BIOMEDAGE Group, Faculty of Medicine, Cantabria University, Santander, Spain

**Keywords:** *Corynebacterium amycolatum*, pan-genome, multidrug resistance, emerging pathogen, virulence factor

## Abstract

*Corynebacterium amycolatum* is a nonlipophilic coryneform which is increasingly being recognized as a relevant human and animal pathogen showing multidrug resistance to commonly used antibiotics. However, little is known about the molecular mechanisms involved in transition from colonization to the MDR invasive phenotype in clinical isolates. In this study, we performed a comprehensive pan-genomic analysis of *C. amycolatum*, including 26 isolates from different countries. We obtained the novel genome sequences of 8 of them, which are multidrug resistant clinical isolates from Spain and Tunisia. They were analyzed together with other 18 complete or draft *C. amycolatum* genomes retrieved from GenBank. The species *C. amycolatum* presented an open pan-genome (*α* = 0.854905), with 3,280 gene families, being 1,690 (51.52%) in the core genome, 1,121 related to accessory genes (34.17%), and 469 related to unique genes (14.29%). Although some classic corynebacterial virulence factors are absent in the species *C. amycolatum*, we did identify genes associated with immune evasion, toxin, and antiphagocytosis among the predicted putative virulence factors. Additionally, we found genomic evidence for extensive acquisition of antimicrobial resistance genes through genomic islands.

## Introduction

Although *Corynebacterium amycolatum* (Collins et al., 1988) is commonly found in the normal microbiome of the human skin and mucosal membranes (Gladysheva et al., 2022), this microorganism is now regarded as a potential multidrug-resistant opportunistic pathogen, especially in nosocomial environments and particularly when it comes to immunocompromised patients (Konstantinidis and Tiedje, 2005; Carvalho et al., 2018; Borde et al., 2020). It has already been described as the causative agent of serious infections in both humans and animals. Focusing on human infections, *C. amycolatum* has been described as the underlaying agent of endocarditis (Konstantinidis and Tiedje, 2005), mastitis (Borde et al., 2020), ear infections (Sengupta et al., 2015), and neonatal sepsis (Berner et al., 1997).

Several studies have shown that *C. amycolatum* infections are often misidentified by culturing and subsequent phenotypic analysis of the isolates, making it difficult to implement appropriate therapeutic interventions (Funke et al., 1996; Zinkernagel et al., 1996; Wauters et al., 1998; Soltan Mohammadi et al., 2013). In this sense, it is essential to define better phenotypic and genetic markers that could improve the identification of pathogenic nonlipophilic members of the genus *Corynebacterium*, including *C. amycolatum* (Santos et al., 2017, 2018). *C. amycolatum* can be clearly distinguished from *C. xerosis* and *C. imitans* by means of MALDI-TOF mass spectrometry using the MALDI Biotyper system (Alibi et al., 2017). However, this technology is not always accessible to all clinical microbiology laboratories, in particular in developing countries. Besides, monitoring the phenotypic profiles of antimicrobial susceptibility is of fundamental importance, as several isolates have demonstrated multiple resistance to antibiotics, in particular to penicillins, clindamycin, aminoglycosides, and fluoroquinolones (Sánchez Hernández et al., 2003; Carvalho et al., 2018; Borde et al., 2020; Dragomirescu et al., 2020).

Previous studies by our group have already demonstrated the potential of comparative genomics to aid the understanding of variability in biochemical reactions commonly used to identify non-diphtherial *Corynebacterium* spp. which are difficult-to-differentiate from *C. amycolatum* in phenotypic tests, particularly *C. xerosis* (Santos et al., 2018). Besides, through comparative genomics we were able to identify specific target genes that can render reliable identification of *C. striatum*, *C. amycolatum* and *C. xerosis* clinical isolates, by multiplex PCR (Santos et al., 2017). More recently, different studies have been demonstrating the added value of whole-genome analyses to improve species circumscription in the genus *Corynebacterium*, including the study by Dover and collaborators (Dover et al., 2021) which proposes a new phylogenomic-based classification of the genus *Corynebacterium*, based on previous studies (Huson and Bryant, 2006), encompassing 19 phylogenetic groups; *C. amycolatum* belongs to the newly proposed group M, that also includes isolates of *C. xerosis* and *C. freneyi* (Dover et al., 2021). Noteworthy, all these previous studies were based on a limited number of isolates of the species *C. amycolatum*. Therefore, an extended pan-genomic analysis of the species can contribute to a better knowledge of the repertoire of gene families, and can aid the understanding of the taxonomy, pathogenicity, lifestyle, and resistome (Moradigaravand et al., 2018; Caputo et al., 2019; Kim et al., 2020).

In this study, we performed a pan-genomic analysis of the species *C. amycolatum*, including genome sequences of 26 isolates from different countries. Eight of these genomic sequences were newly generated in this work and were derived from clinical isolates of *C. amycolatum* from Spain and Tunisia, which presented multiple resistance to antimicrobial agents (Supplementary Tables S1, S2). Therefore, we can infer that the species *C. amycolatum* has an open pan-genome, with major horizontal acquisition of antimicrobial resistance genes through genomic islands and many virulence factors.

## Materials and methods

### Whole-genome sequencing of new clinical isolates and retrieval of *Corynebacterium amycolatum* genomic sequences from public databases

Next-generation sequencing was performed for eight new clinical isolates, which were identified as *C. amycolatum / xerosis* by the API Coryne biochemical battery and by MALDI-ToF mass spectrometry, according to standard protocols: strains FA111 and FA86 isolated at Farhat Hached Hospital (Sousse, Tunisia); strains VH1773, VH2077, VH2225, VH4147_1, VH4147_3, and VH6958 isolated at University Hospital Marqués de Valdecilla (Santander, Spain; please, see Supplementary Tables S1, S2 for clinical information and antimicrobial susceptibility profiles of the isolates). The isolates were cultured on blood agar plates for 48 h at 37°C, and the genomic DNA was extracted using the NucleoSpin Microbial DNA Kit (Macherey-Nagel). For next-generation sequencing using the Illumina HiSeq 2,500 platform (Illumina Inc.), sequencing libraries were prepared by the NEBNext® Fast DNA Fragmentation and Library Preparation Kit for Illumina® (New England Biolabs Inc.), as previously described (Rocha et al., 2020). Genome sequences were obtained for paired-end libraries with a minimum coverage of 1,000x. Genomic assemblies were obtained through the automated pipeline available at the PATRIC platform (Wattam et al., 2017) using SPAdes (Bankevich et al., 2012).

Eighteen additional genomic sequences for the species *C. amycolatum* (complete or draft) were retrieved from the National Center for Biotechnology Information (NCBI)’s GenBank (Tatusova et al., 2016).

### Average nucleotide identity (ANIb) and TETRA

To certify that the genomic sequences are circumscribed within the *C. amycolatum* species, we performed average nucleotide identity by BLAST (ANIb) and tetranucleotide signature (TETRA) analyses through the JSpeciesWS platform (Richter et al., 2016).

### Pan-genomic analysis

For standardization, all assembled genomic sequences were annotated using NCBI’s Prokaryotic Genome Annotation Pipeline (PGAP; Tatusova et al., 2016). Pan-genomic analysis was performed with the Bacterial Pan Genome Analysis (BPGA 1.3) tool (Chaudhari et al., 2016), using a 50% identity cut-off and the USEARCH pipeline for gene grouping (Edgar, 2010). BPGA uses the Power Law regression model (*n* = *k*. *N*^α^) to determine whether the pan-genome is open (*α* ≤ 1) or closed (*α* > 1; Tettelin et al., 2005, 2008).

### Functional annotations

The subgroups of the pan-genome were submitted for annotation of the Cluster of Orthologous Groups (COG) functional categories using the eggNOG-Mapper (Huerta-Cepas et al., 2017). The prediction of antibiotic resistance genes was performed in the Pathosystems Resource Integration Center (PATRIC) platform (Wattam et al., 2017) using the Comprehensive Antibiotic Resistance Database (CARD; Jia et al., 2017) and Database of Antibiotic-Resistant Organisms (NDARO).^1^ Virulence factors were evaluated through VFanalyzer and the Virulence Factor Database (VFDB; Liu et al., 2019). The key genes involved in the mycolic acid biosynthetic pathway were searched in the *C. amycolatum* genomes using the sequences and the method described by Dover and collaborators (Dover et al., 2021).

### Predictions of genomic Islands, phages, and plasmid-derived sequences

IslandViewer 4 (Bertelli et al., 2017) was used for genomic islands prediction by integrating IslandPath-DIMOB (Hsiao et al., 2003), IslandPick (Langille et al., 2008), SIGI-HMM (Waack et al., 2006), and Islander (Hudson et al., 2015). Circular plots of the genomic sequences were plotted using BLAST Ring Image Generator (BRIG), including reference positions for antimicrobial resistance genes (AMR), virulence factors (VF), and genomic islands (GI; Alikhan et al., 2011).

Phage sequences were predicted with the Phage Search Tool Enhanced Release (PHASTER) platform (Arndt et al., 2016), which has approximately 187,000 phage sequences in the database. Plasmid searches were performed with the PlasmidFinder platform (Carattoli et al., 2014), which searches for plasmid replicons, and with the RFPlasmid platform (van der Graaf-van Bloois et al., 2021), which identifies plasmid sequences in contigs generated from short-read sequencing, by searching for specific proteins and plasmid replicons.

### Deposit of genomic sequences in public databases

The genomic sequences generated in this study are publicly accessible through NCBI’s GenBank, with the respective accession numbers: JAFJMB000000000, JAFJMC000000000, JAFJMD000000000, JAFJME000000000, JAFJMF000000000, JAFJMG000000000, JAFJMH000000000, and JAFJMI000000000. A detailed description of the genomes can be found in Supplementary Table S1.

## Results and discussion

### General features of the *Corynebacterium amycolatum* genomes

Among the 26 *C. amycolatum* studied genomes, five were marked as complete genomes, and the remaining are in draft versions (see Supplementary Table S1). The estimated genome sizes range between 2.42 and 2.82 Mbp, with the G + C% content varying less than 1% between the isolates (58.6–59.0%). The numbers of annotated coding sequences (CDS) ranges from 2,038 (for isolate UMB1182) to 2,371 (for isolate FDAARGOS_991; see Supplementary Table S1).

**Figure 1:**
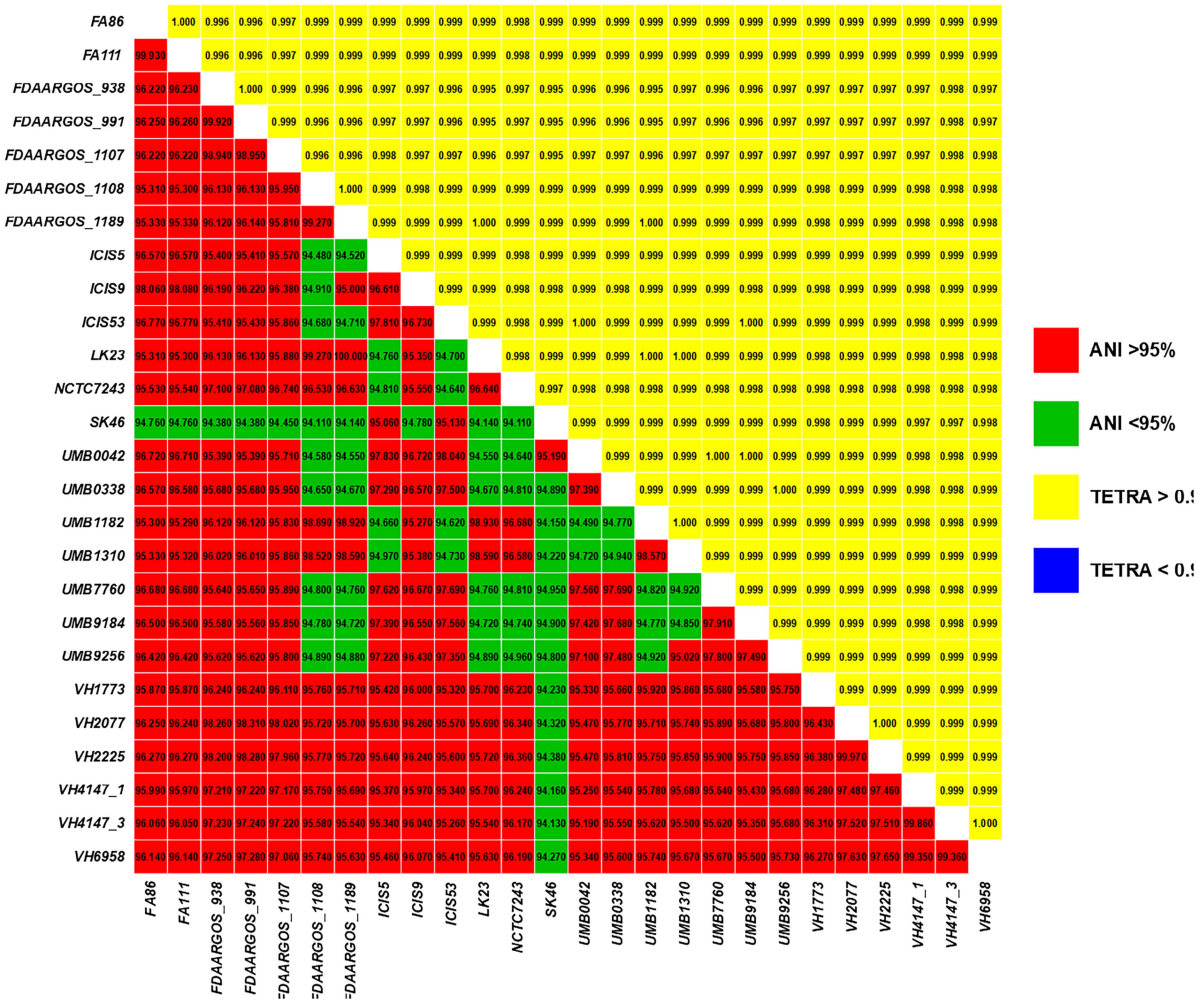
Pairwise comparisons of ANIb values (bottom triangle) and TETRA values (upper triangle) between all studied *C. amycolatum* genomic sequences.

The species assignment of the genomic sequences through ANIb (Figure 1) showed that the SK46 isolate was below the generally regarded cutoff value for species delineation (95.0%) when compared with other *C. amycolatum* isolates: identity between the SK46 and the NCTC7243 strain was 94.11%. Nevertheless, it has been described that the values above 94.0% are equivalent to 70% of DNA–DNA hybridization (DDH), a method considered as the gold standard for species identification (Richter and Rosselló-Móra, 2009). The results of the TETRA analysis were approximately 0.999, and the variation of the percentage of GC was ≤1%, reinforcing that the SK46 lineage is circumscribed within the species *C. amycolatum* (Figure 1).

### Pan-genomic analysis and functional annotations by COGs

**Figure 2:**
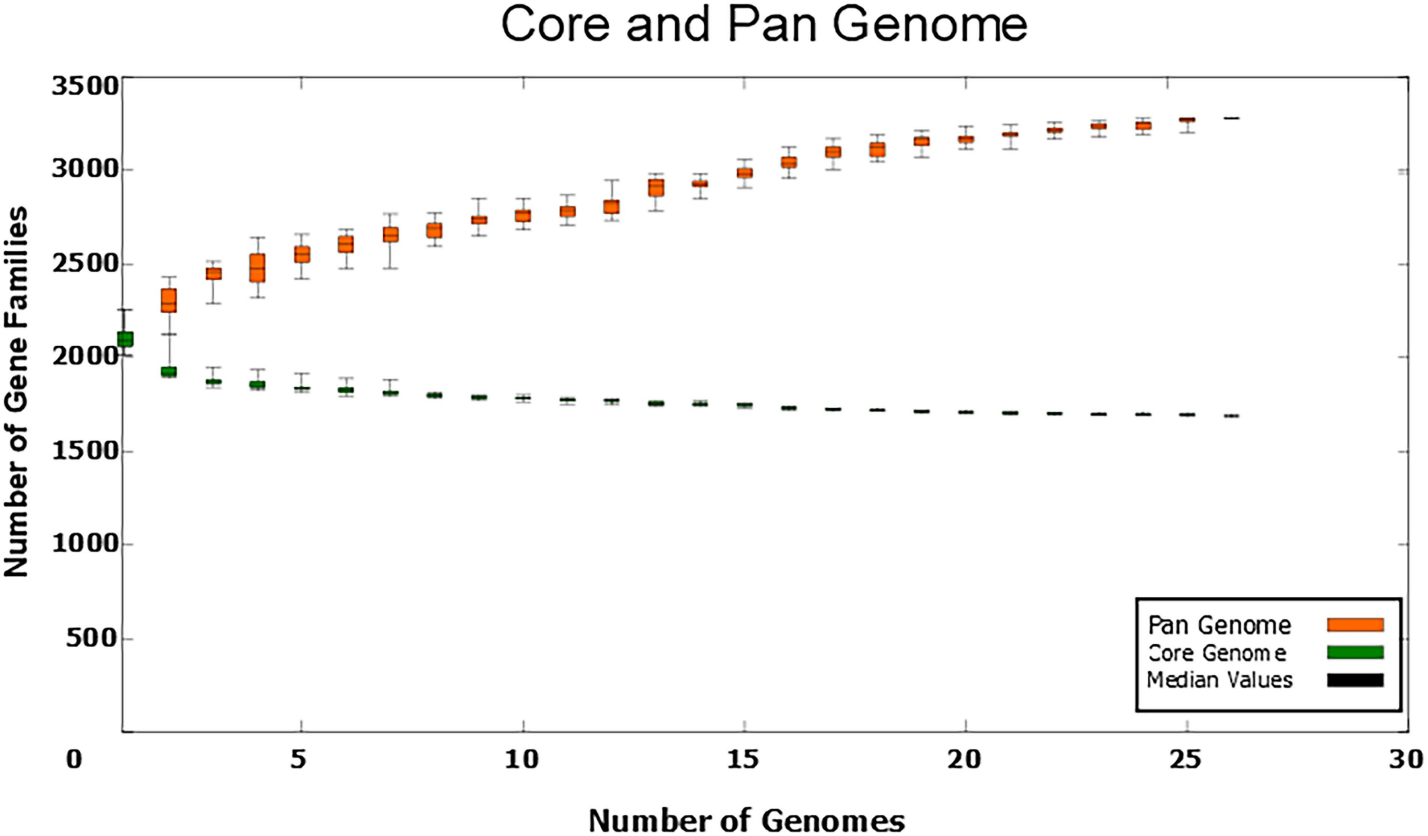
Numbers of gene families in the *C. amycolatum* pan-genome vs. numbers of new genomes added to the analysis.

**Figure 3:**
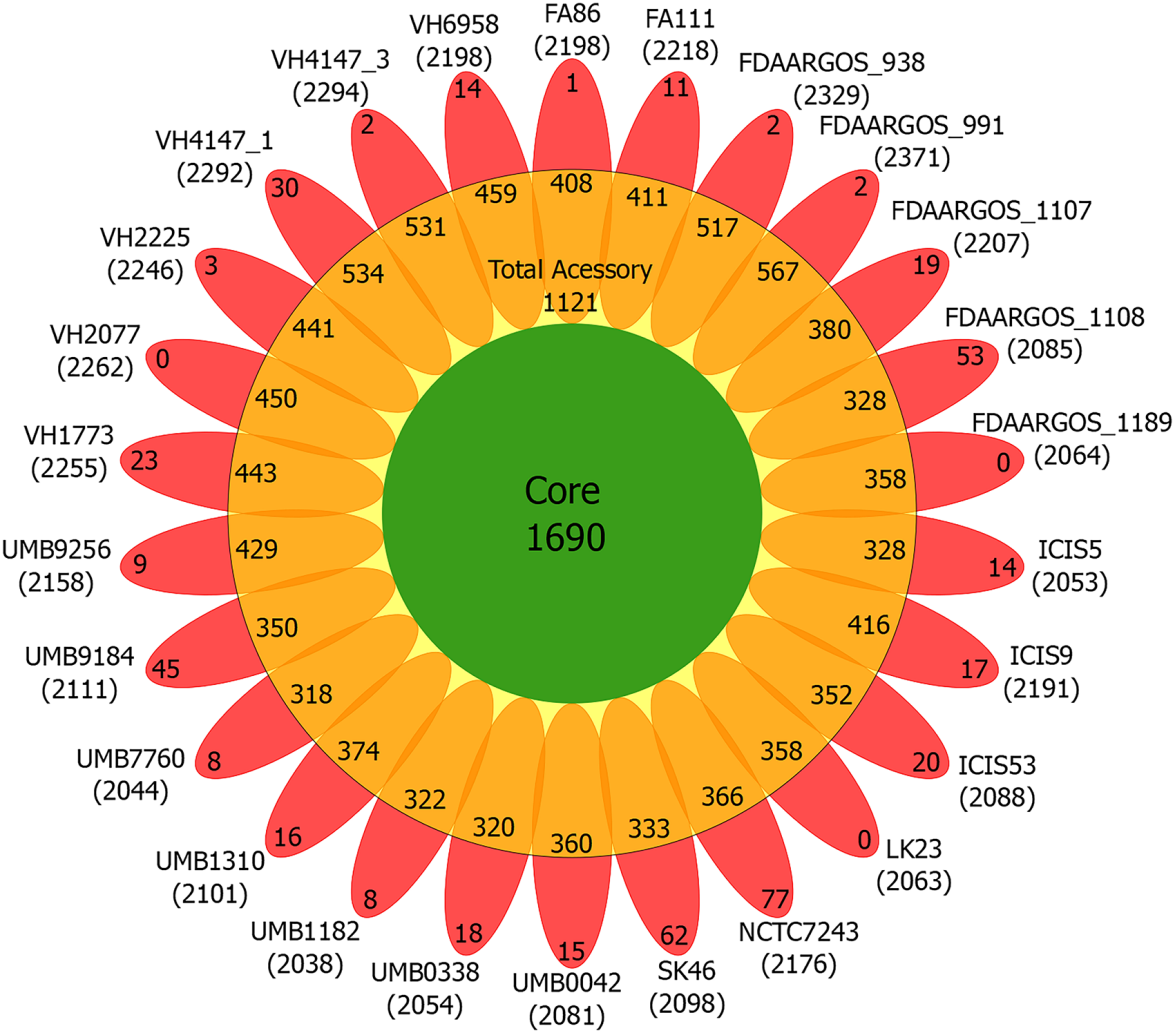
Flower plot diagram of the *C. amycolatum* pan-genome, showing core gene families (green), accessory genes present in each isolate (orange), and unique genes per genome (red). Within parentheses are the numbers of predicted coding sequences for each isolate.

The *C. amycolatum* pan-genome has 3,280 predicted gene families (Figure 2), being 1,690 in the core genome (51.52%), 1,121 related to accessory genes (34.17%), and 469 related to unique genes (14.29%; Figure 3). The estimated α value of 0.854905 indicates an open pan-genome, and the predicted core genome stabilizes with *approx.* 1,641 gene families.

**Figure 4:**
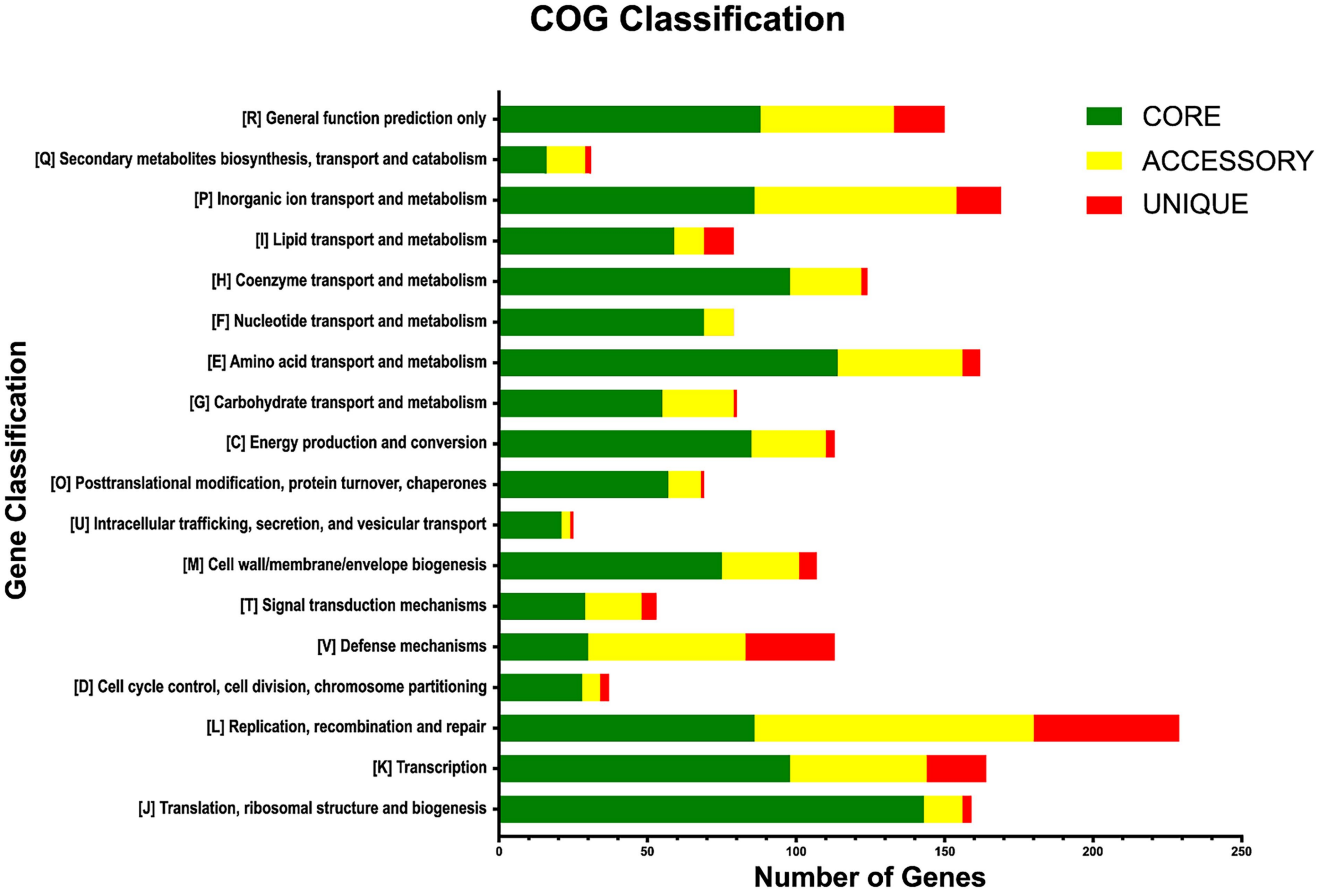
Functional annotations of the pan-genome subsets according to COG categories.

The functional annotations of the pan-genomic subsets revealed that the core genome and accessory gene families are primarily classified in the ‘Metabolism’ category, with 582 and 216 annotated gene families (34.0 and 19.0%), respectively. Unique genes were mainly ranked in the ‘Information storage and processing’ category, with 72 genes in this class (15.0%). The main COG subcategories in the core genome were: translation, ribosomal structure and biogenesis (8.5%); amino acid transport and metabolism (6.7%); coenzyme transport and metabolism (5.8%); transcription (5.8%); and inorganic ion transport and metabolism (5.1%; Figure 4). Accessory genes were mainly involved in functions of replication, recombination, and repair (8.4%); inorganic ion transport and metabolism (6.1%); defense mechanisms (4.7%); transcription (4.1%); and amino acid transport and metabolism (3.7%). Unique genes were mostly related to biological functions of replication, recombination, and repair (10.4%); defense mechanisms (6.4%); transcription (4.3%); inorganic ion transport and metabolism (3.2%); and lipid transport and metabolism (2.1%). A total of 1,337 genes were labeled as ‘unknown function genes’, comprising 453 genes (26.8%) in the core genome, 589 (52.54%) in accessory genes, and 295 (62.89%) in the unique genes group (Figure 4).

### Prediction of antimicrobial resistance genes and genomic islands

**Figure 5:**
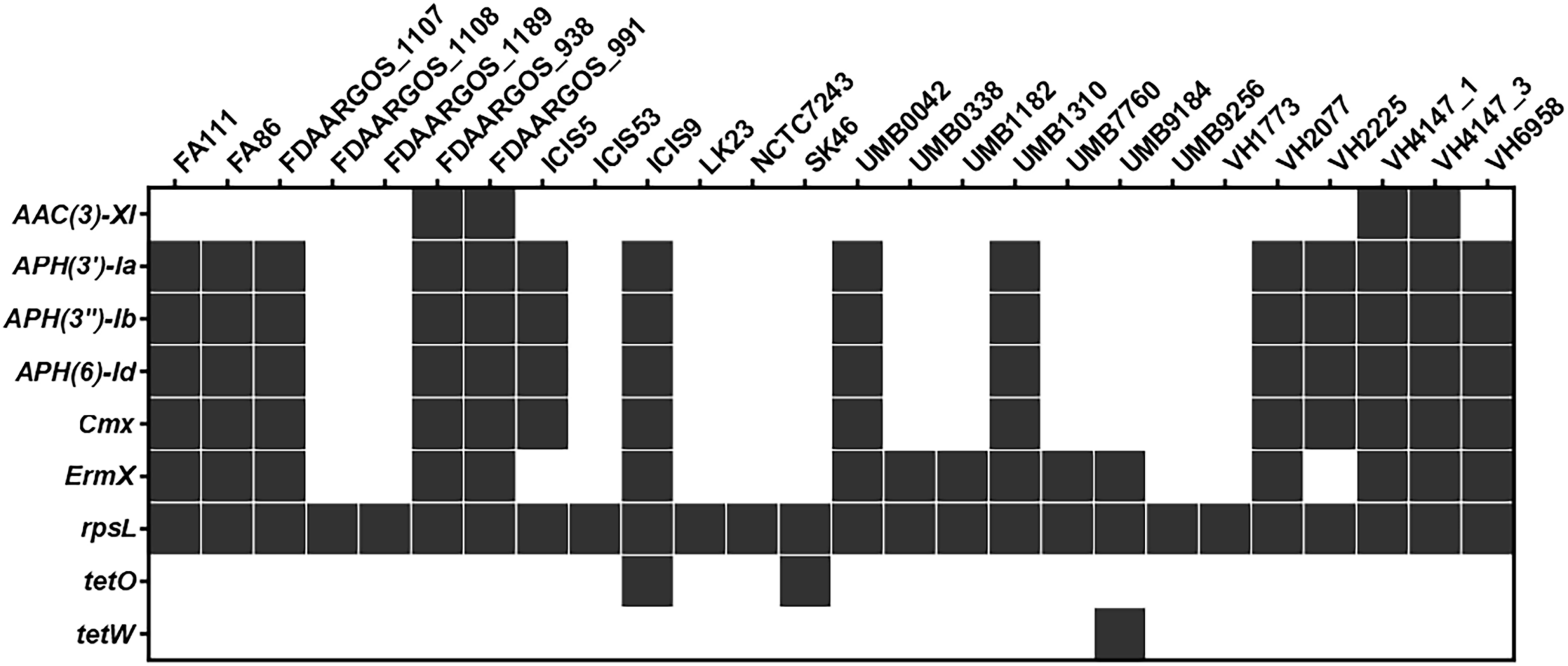
The *C. amycolatum* resistome predicted through automated annotation in the PATRIC platform.

The PATRIC platform identified nine antimicrobial resistance genes (AMRs) by automatic annotation of the *C. amycolatum* resistome (Figure 5). Only the *rpsL* gene was identified in all studied strains, containing mutations similar to those detected in streptomycin-resistant *Mycobacterium tuberculosis* isolates (Sreevatsan et al., 1996). Seven AMRs were placed in the accessory genome of *C. amycolatum*, which confer resistance to aminoglycosides, chloramphenicol, streptogramins, macrolides, lincosamides, and tetracycline: *aac(3)-XI* (aminoglycoside 3-N-acetyltransferase), identified in 15.0% of genomes; *aph(3′)-Ia*, *aph(3″)-Ib*, *aph(6)-Id* (aminoglycoside phosphotransferases), in 54% of isolates; *cmx* (efflux pump major facilitator superfamily, MFS), in 54% of isolates; *ermX* (Erm 23S ribosomal RNA methyltransferase), in 62% of isolates; and *tetO* (tetracycline resistance) in only 2 isolates. The *tetW* gene, encoding a ribosomal protection protein, was the single AMR gene found as unique in the pan-resistome of *C. amycolatum*, being detected only in the genomic sequence of isolate UMB9184 (Figure 5).

**Figure 6:**
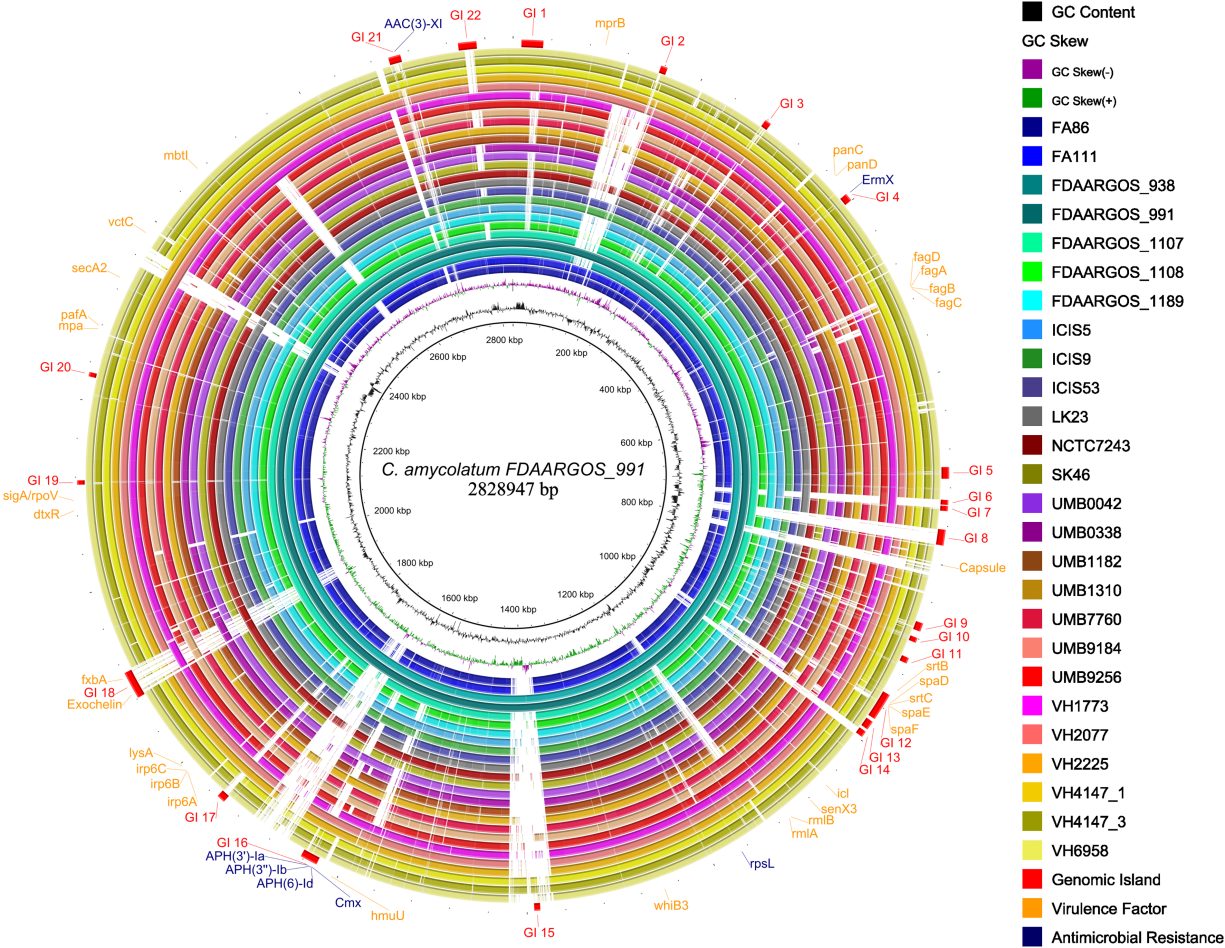
Circular genome plot showing the distribution of predicted genomic islands (GIs) in the studied *C. amycolatum* genomes. Most outer circle presents the positions of GIs and respective AMR genes. The genome sequence of the isolate FDAARGOS_911 was used as a reference.

Apart from the *rpsL* gene, all other predicted AMRs co-localize with predicted genomic islands in the studied genomes (Figure 6). The genes *cmx*, *aph(3′)-Ia*, *aph(3″)-Ib*, *aph(6)-Id* were consistently found within the exact genomic location (Figure 6), indicating a common mechanism of horizontal acquisition of AMR genes.

### Phages and plasmid-associated sequences in *Corynebacterium amycolatum*

The phage prediction detected 38 sequences (Supplementary Figure S1), the most frequent was the *Corynebacterium* Juicebox phage, present in 15 strains (Supplementary Figure S2), followed by the *Corynebacterium* phage SamW, identified in 6 strains. In total, 13 different phages were found. We identified the *ermX* gene within the Gordon phage Daredevil sequence in the *C. amycolatum* lineage UMB9184. The results generated by the RFPlasmid tool identified 36 sequences containing plasmid signatures among the studied strains, in which 27 AMR genes were present (Supplementary Table S2). This represents approximately 26% of the total predicted AMR genes. The ICIS5, ICIS9, VH2225, VH4147_1, and VH4147_3 strains presented sequences containing similar context with the AMR genes *cmX*, *aph (6)-Id*, *aph(3″)-Ib*, and *aph(3′)-Ia*. Analyzes performed with PlasmidFinder, however, did not detect any plasmid-related sequences, when searching for plasmid replicons.

### Potential virulence factors

**Figure 7:**
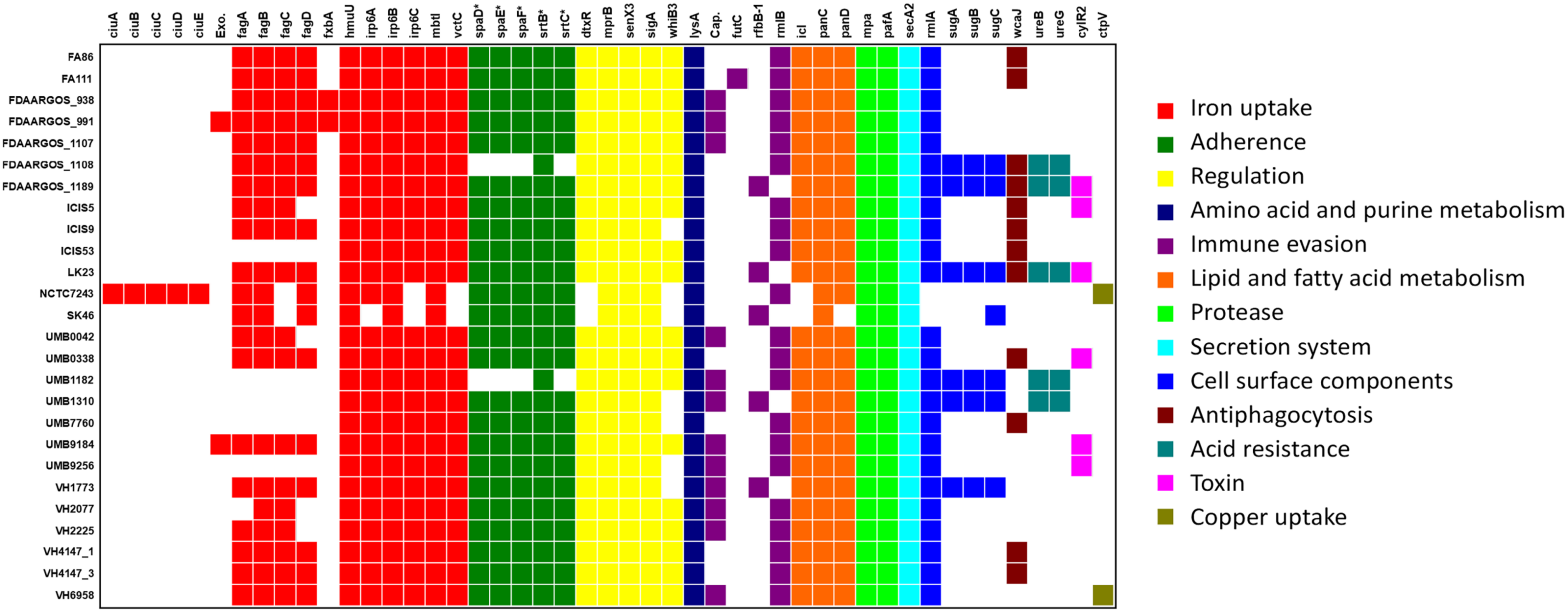
Distribution of virulence factors throughout the *C. amycolatum* genomic sequences. According to the legend, colors indicate the functional classes of the identified genes. Exo = exochelin coding sequence (ortholog to *M. smegmatis* gene); Cap = capsule related gene (ortholog to *Acinetobacter* spp). * Denotes gene sequences annotated by manual curation after automatic prediction.

Forty-seven genes were found in the *C. amycolatum* pan-genome, which can be potentially associated with virulence functions (Figure 7). The majority of these virulence genes are present in the accessory genome (29 genes), while 12 genes are shared by all strains (core genome), and only 6 genes appear as unique to single isolates (Figure 7). Genes involved in iron acquisition are particularly enriched in this category of potential virulence genes, with 17 of those genes found in the species *C. amycolatum*. The operon *ciuABDE,* coding for an ABC-type siderophore transporter system (Kunkle and Schmitt, 2005), was found only in the strain NCTC7243. The *fagABCD* operon, coding for iron-siderophore transport through the membrane (Billington et al., 2002), was located entirely in 15 *C. amycolatum* genomes and partially found in additional 5 genomes. Twenty-four genomes also presented genes coding for the complete heterodimeric transporter *Irp6ABC* (Qian et al., 2002), while 2 genomes showed an incomplete coding potential. Additionally, the gene *hmuU* involved in the heme-transporter system *hmuTUV* of *C. diphtheriae* and *C. ulcerans* (Drazek et al., 2000) was found in all *C. amycolatum* genomes. An ortholog of the *vctC* gene that is part of the *vctPDGC* heme-transportation system in *Vibrio cholerae* (Wyckoff and Payne, 2011) was also found in 24 *C. amycolatum* genomes. Regarding siderophore biosynthesis pathways, we found orthologs in all studied genomes for the genes *mbtI* from *Mycobacterium tuberculosis* (Mori et al., 2020) and *fxbA* from *Mycobacterium smegmatis*. The latter is part of the biosynthetic pathway of mycobacterial exochelin (lipid- and water-soluble siderophore; Ojha and Hatfull, 2007) and was only found in two *C. amycolatum* isolates (FDAARGOS_938 and FDAARGOS_991).

Gene sequences coding for SpaD-like pili were predicted in most *C. amycolatum* isolates (Figure 7). In this adherence machinery, the proteins SpaD, SpaE, and SpaF form a filamentous structure that remains anchored to the bacterial surface and needs the sortases SrtB and SrtC for the anchoring step (Gaspar and Ton-That, 2006). These adherence structures are involved in essential pathogenicity functions that include host tissue colonization (Swaminathan et al., 2007), adherence under mechanical stress conditions (Echelman et al., 2016), and biofilm biogenesis (Swaminathan et al., 2007).

All *C. amycolatum* genomes presented genes coding for a functional ATP-dependent proteasome system, namely *mpA* (*Mycobacterium* proteasome ATPase) and *pafA* (proteasome accessory factor A; Pearce et al., 2008). Interestingly, six *C. amycolatum* genomes presented the *cylR2* gene, whose product acts as a repressor of the cytolysin operon in *Enterococcus faecalis* (Haas et al., 2002).

The virulence genes *fxbA*, *exc* (exochelin), and the operons *ciuABDE, sugABC, spaDEF* plus the sortase genes *srtB* and *srtC* were mainly predicted within the context of genomic islands, showing their role in horizontal acquisition of variable genes.

These results were obtained from the VFDB database, which gathers information about virulence factors from studies that evaluated the ability of mutants to develop disease in the host (Liu et al., 2022). In this sense, our results reinforce the relevance of genes coding for *pili* (Broadway et al., 2013; Oliveira et al., 2017) and siderophores (Kunkle and Schmitt, 2003; Ibraim et al., 2019) in the *Corynebacterium* genus. Importantly, some studies have already discussed the important roles these genes play not only in virulence, but also in adaptation to distinct niches (Swierczynski and Ton-That, 2006; Tauch and Burkovski, 2015). Although we did not identify classic corynebacterial virulence factors in *C. amycolatum*, which are commonly associated with known pathogens of the *Corynebacterium* genus, such as diphtheria toxin, phospholipase D, and hemolysins (Dorella et al., 2006; Parveen et al., 2019), we were able to detect genes associated with immune evasion, antiphagocytosis, and toxins, that may be relevant to the pathogenicity of this species.

Mycolic acids are essential components of the cell wall of most Actinobacteria (Collins et al., 1982; Ioneda, 1993). They play a crucial role in the interaction of *M. tuberculosis* with host cells (Korf et al., 2005). However, *C. amycolatum* lacks corynomycolic acids in its cell wall (Barreau et al., 1993). The search for key genes of the mycolic acid biosynthesis pathway in *C. amycolatum* showed the absence of the essential genes (Supplementary Figure S3), especially the *fadD32-pks13-accD4* operon (Portevin et al., 2005; Gavalda et al., 2009) and the *cmrA* gene (Lea-Smith et al., 2007) involved in mycolic acid condensation, then confirming that the species *C. amycolatum* does not have the genetic potential to synthesize mycolic acids; these findings corroborate the results from previous genomic studies of *C. amycolatum* (Daffé and Draper, 1997; Daffé, 2005; Baek et al., 2018; Dover et al., 2021).

## Conclusion

The *C. amycolatum* pan-genome demonstrated an open status, which corroborates the high number of predicted genomic islands containing antimicrobial resistance genes (AMRs) and sequences coding for potential virulence factors. These biological functions are mainly acquired through horizontal gene transfer in the species. Notably, the high number of horizontally-acquired virulence genes that code for functions related to adaptation to the host organism, such as iron acquisition and adherence, may aid in the understanding of the pathogenic potential of this generally-regarded as commensal microorganism. In addition, the fact that we identified a genomic island containing genes that confer resistance to aminoglycosides and chloramphenicol in more than 50% of the studied isolates demonstrates the importance of unambiguous identification of this potentially pathogenic microorganism by clinical microbiology laboratories.

## Data availability statement

The datasets presented in this study can be found in online repositories. The names of the repository/repositories and accession number(s) can be found in the article/Supplementary material.

## Author contributions

HJ, DR, EA, and LP: investigation, formal analysis, methodology, software, data curation, visualization, and writing – original draft. RR, AS, BB, AG-N, MC, SS, VA, LM-M, AO, and SA: resources, project administration, and writing – review and editing. LP and JN: conceptualization, funding, project administration, supervision, and writing – review and editing. All authors contributed to the article and approved the submitted version.

## Funding

This study was partially supported by grants from FAPESB, CNPq, CAPES, FINEP, and RECOM Network, in Brazil. HJ was recipient of a PhD scholarship from FAPESB. LP was recipient of a research fellowship from CNPq.

## Conflict of interest

The authors declare that the research was conducted in the absence of any commercial or financial relationships that could be construed as a potential conflict of interest.

## Publisher’s note

All claims expressed in this article are solely those of the authors and do not necessarily represent those of their affiliated organizations, or those of the publisher, the editors and the reviewers. Any product that may be evaluated in this article, or claim that may be made by its manufacturer, is not guaranteed or endorsed by the publisher.

## Supplementary material

The Supplementary material for this article can be found online at: https://www.frontiersin.org/articles/10.3389/fmicb.2022.1011578/full#supplementary-material

## Footnotes

^1^ https://www.ncbi.nlm.nih.gov/pathogens/antimicrobial-resistance/
