# Supplementary material for: Pan-genomic analysis of Corynebacterium amycolatum gives insights into molecular mechanisms underpinning the transition to a pathogenic phenotype
Source: Front Microbiol. 2022 Nov 16;13:1011578. doi: 10.3389/fmicb.2022.1011578 (PMC9709149; doi:10.3389/fmicb.2022.1011578)

**SUPPLEMENTARY MATERIAL**

Pan-genomic analysis of *Corynebacterium amycolatum* gives insights into molecular mechanisms underpinning the transition to a pathogenic phenotype

Hendor N. R. Jesus^1^, Danilo J. P. G. Rocha^2^, Rommel T. J. Ramos^3^, Artur Silva^3^, Bertram Brenig^4^, Aristóteles Góes-Neto^5^, Mateus M. Costa^6^, Siomar C. Soares^7^, Vasco Azevedo^5^, Eric R. G. R. Aguiar^8^, Luis Martínez-Martínez^9,10,11^, Alain Ocampo^12^, Sana Alibi^13^, Alexis Dorta^14^,Luis G. C. Pacheco^1,2,*^, Jesus Navas^14*^

^1^ Multicenter Post-Graduate Program in Biochemistry and Molecular Biology (PMBqBM), Institute of Health Sciences, Federal University of Bahia, Salvador-BA, Brazil

^2^ Post-Graduate Program in Biotechnology, Institute of Health Sciences, Federal University of Bahia, Salvador-BA, Brazil

^3^ Institute of Biological Sciences, Federal University of Para, Belém-PA, Brazil

^4^ University of Göttingen, Institute of Veterinary Medicine, Göttingen, Germany

^5^ Institute of Biological Sciences, Federal University of Minas Gerais, Belo Horizonte-MG, Brazil

^6^ Universidade do Vale do São Francisco, Petrolina, Pernambuco, Brazil.

^7^ Universidade Federal do Triângulo Mineiro-UFTM, Uberaba, Minas Gerais, Brazil.

^8^ Department of Biological Sciences, State University of Santa Cruz, Ilhéus-BA, Brazil

^9^ Unidad de Gestión Clínica, Hospital Universitario Reina Sofía, Av. Menéndez Pidal, s/n, 14004 Córdoba, Spain. luis.martinez.martinez.sspa@juntadeandalucia.es

^10^ Departamento de Microbiología, Universidad de Córdoba, Campus Rabanales. Edif. Severo Ochoa (C6), 14071 Córdoba, Spain

^11^ Instituto Maimónides de Investigación Biomédica de Córdoba (IMIBIC), Av. Menéndez Pidal, s/n, 14004 Córdoba, Spain

^12^ Microbiology Service. University Hospital Marqués de Valdecilla, Santander, Spain. Instituto de Investigación Valdecilla (IDIVAL), Santander, Spain

^13^ Research Unit Analysis and Process Applied to the Environment (UR17ES32), Tunisia

^14^ BIOMEDAGE Group. Faculty of Medicine. Cantabria University, Santander, Spain; Instituto de Investigación Valdecilla (IDIVAL), Santander, Spain

Running Title: The *Corynebacterium amycolatum* Pan-Genome

*** Correspondence:**Jesús Navas
[navasj@unican.es](mailto:navasj@unican.es)

**Supplementary Table S1.** Information on *C. amycolatum* isolates, patients, and source of isolation.

| Strain | Patient information and isolation source |
| --- | --- |
| C. amycolatum FA111 | Isolated from a 70-year-old diabetic woman with foot infection hospitalized in the Endocrinology service. A swab was taken from an abscess produced in a toe. |
| C. amycolatum FA86 | Isolated from a 48-year-old female patient hospitalized in the Hematology Department for recurrent bone marrow aplasia. She had otorrhea and the bacteria was isolated as pure culture from a pus swab taken from the ear. |
| C. amycolatum VH1773 | Isolated as a pure culture from a surgical wound exudate swab taken from a patient presenting with skin and soft-tissue infection. |
| C. amycolatum VH2077 | Isolated after culturing of a pus swab from an abdominal drainage from a patient with abdominal infection diagnosed of biliary peritonitis. |
| C. amycolatum VH2225 | Isolated as a pure culture from a swab of perianal abscess taken from a patient presenting with skin and soft-tissue infection (Fournie’s gangrene). |
| C. amycolatum VH4147_1 | Isolated from a percutaneous endoscopic gastrostomy exudate swab from a patient with skin and soft-tissue infection. |
| C. amycolatum VH4147_3 | Is a morphotype of VH4147_1. |
| C. amycolatum VH6958 | Isolated as pure culture from a cervicofacial swab taken from a patient diagnosed of jaw osteomyelitis. |

All strains identified as *C. amycolatum* were isolated as pure culture from swabs of patients presenting with skin and soft tissue infection.

**Supplementary Table S2.** Antimicrobial susceptibility profile of C. amycolatum isolates.

| Strain | Penicillin | Gentamicin | Erythromycin | Ciprofloxacin | Rifampicin | Linezolid | Vancomycin |
| --- | --- | --- | --- | --- | --- | --- | --- |
| VH1773 | R | S | R | R | S | S | S |
| VH2225 | R | S | S | R | S | S | S |
| VH2077 | R | S | R | R | S | S | S |
| VH4147_1 | R | S | R | R | S | S | S |
| VH4147_3 | R | S | R | R | S | S | S |
| FA86 | R | R | R | R | R | S | S |
| FA111 | R | R | R | S | S | S | S |

Method: Disk diffusion (EUCAST standardized disk diffusion method)
Medium: Mueller-Hinton agar + 5% defibrinated horse blood and 20 mg/L β-NAD

Quality control: *Streptococcus pneumoniae* ATCC 49619

Defined as R or S according to EUCAST Clinical Breakpoints Tables v. 12.0, Zone diameter breakpoints (mm) ([www.eucast.org](http://www.eucast.org))

**Supplementary Table S3.** General features about the *C. amycolatum* genomic sequences used in this study

| Strain | Assembly | Status | Size | GC% | Contigs | CDS |
| --- | --- | --- | --- | --- | --- | --- |
| *FA111* | GCA_024539885.1 | Contig | 2594980 | 58.8 | 133 | 2218 |
| *FA86* | GCA_024539865.1 | Contig | 2578559 | 58.9 | 124 | 2198 |
| *FDAARGOS_1107* | GCA_016728745.1 | Complete | 2706528 | 58.7 | 1 | 2207 |
| *FDAARGOS_1108* | GCA_016728725.1 | Complete | 2476115 | 58.6 | 1 | 2085 |
| *FDAARGOS_1189* | GCA_016889425.1 | Complete | 2474943 | 58.6 | 1 | 2064 |
| *FDAARGOS_938* | GCA_016026415.1 | Complete | 2802624 | 58.6 | 1 | 2329 |
| *FDAARGOS_991* | GCA_016127615.1 | Complete | 2828947 | 58.6 | 1 | 2371 |
| *ICIS 5* | GCA_008368695.1 | Contig | 2474151 | 58.8 | 115 | 2053 |
| *ICIS 53* | GCA_001722255.1 | Contig | 2460257 | 59.0 | 41 | 2088 |
| *ICIS 9* | GCA_001975925.1 | Contig | 2587830 | 58.6 | 181 | 2191 |
| *LK23* | GCA_014335175.1 | Contig | 2451386 | 58.7 | 10 | 2063 |
| *NCTC7243* | GCA_900461385.1 | Contig | 2638280 | 58.8 | 6 | 2176 |
| *SK46* | GCA_000173655.1 | Contig | 2513912 | 58.6 | 48 | 2098 |
| *UMB0042* | GCA_002847785.1 | Scaffold | 2481374 | 58.9 | 16 | 2081 |
| *UMB0338* | GCA_002861405.1 | Scaffold | 2465364 | 58.9 | 39 | 2054 |
| *UMB1182* | GCA_008726785.1 | Contig | 2425544 | 58.7 | 41 | 2038 |
| *UMB1310* | GCA_008726645.1 | Contig | 2492575 | 58.8 | 54 | 2101 |
| *UMB7760* | GCA_008726455.1 | Contig | 2422276 | 58.9 | 65 | 2044 |
| *UMB9184* | GCA_008726215.1 | Contig | 2494409 | 58.9 | 61 | 2111 |
| *UMB9256* | GCA_008726175.1 | Contig | 2565104 | 58.8 | 65 | 2158 |
| *VH1773* | GCA_024539915.1 | Contig | 2641344 | 58.9 | 103 | 2255 |
| *VH2077* | GCA_024539855.1 | Contig | 2642041 | 58.6 | 134 | 2262 |
| *VH2225* | GCA_024539935.1 | Contig | 2632447 | 58.7 | 130 | 2246 |
| *VH4147_1* | GCA_024539835.1 | Contig | 2664651 | 58.7 | 145 | 2292 |
| *VH4147_3* | GCA_024539775.1 | Contig | 2639734 | 58.8 | 142 | 2294 |
| *VH6958* | GCA_024539755.1 | Contig | 2590091 | 58.8 | 93 | 2198 |

**Supplementary Table S4.** AMR genes and predicted plasmid-associated proteins in contigs classified as being plasmid-derived in *C. amycolatum* genomes.

| **Strains** | **AMR Genes** | **# of Plasmid Genes** |
| --- | --- | --- |
| FA86 | *aph(3')-Ia* | 11 |
| ICIS5 | *cmx* | 6 |
|  | *aph(6)-Id* |  |
|  | *aph(3'')-Ib* |  |
|  | *aph(3')-Ia* |  |
| ICIS9 | *aph(3'')-Ib* | 14 |
|  | *aph(6)-Id* |  |
|  | *cmx* |  |
|  | *tetO* |  |
|  | *aph(3')-Ia* |  |
| SK46 | *tetO* | 1 |
| UMB0042 | *aph(3')-Ia* | 1 |
| UMB1310 | *aph(3')-Ia* | 5 |
| UMB9184 | *tetW* | 2 |
| VH2077 | *ErmX* | 6 |
| VH2225 | *cmx* | 9 |
|  | *aph(6)-Id* |  |
|  | *aph(3'')-Ib* |  |
|  | *aph(3')-Ia* |  |
| VH4147_1 | *aph(3'')-Ib* | 11 |
|  | *aph(6)-Id* |  |
|  | *cmx* |  |
|  | *aph(3')-Ia* |  |
| VH4147_3 | *aph(3'')-Ib* | 12 |
|  | *aph(6)-Id* |  |
|  | *cmx* |  |
|  | *aph(3')-Ia* |  |
| FA111 | No predicted gene | 5 |

**Supplementary Figure S1.** Distribution of phage sequences detected in the *C. amycolatum* genomes.

**
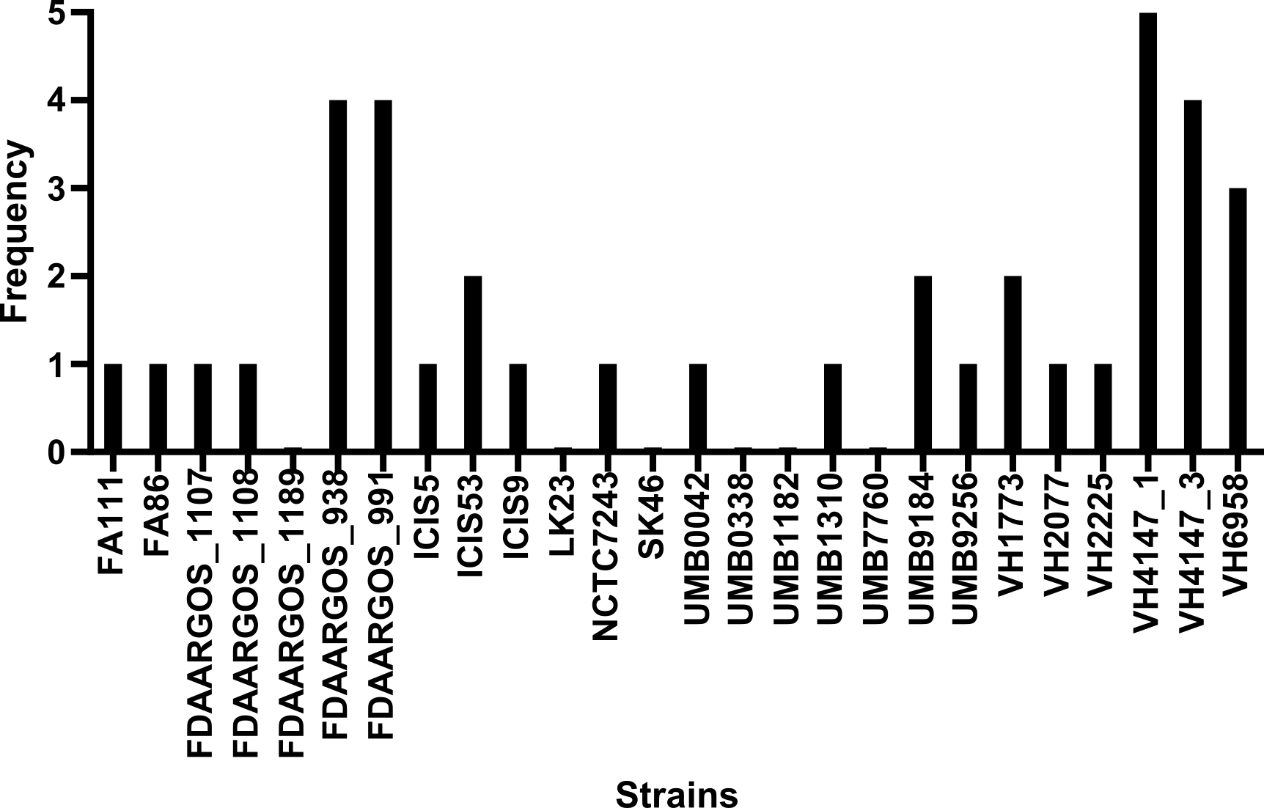
**

**Supplementary Figure S2.** Frequencies of phages detected in the *C. amycolatum* genomes.

**
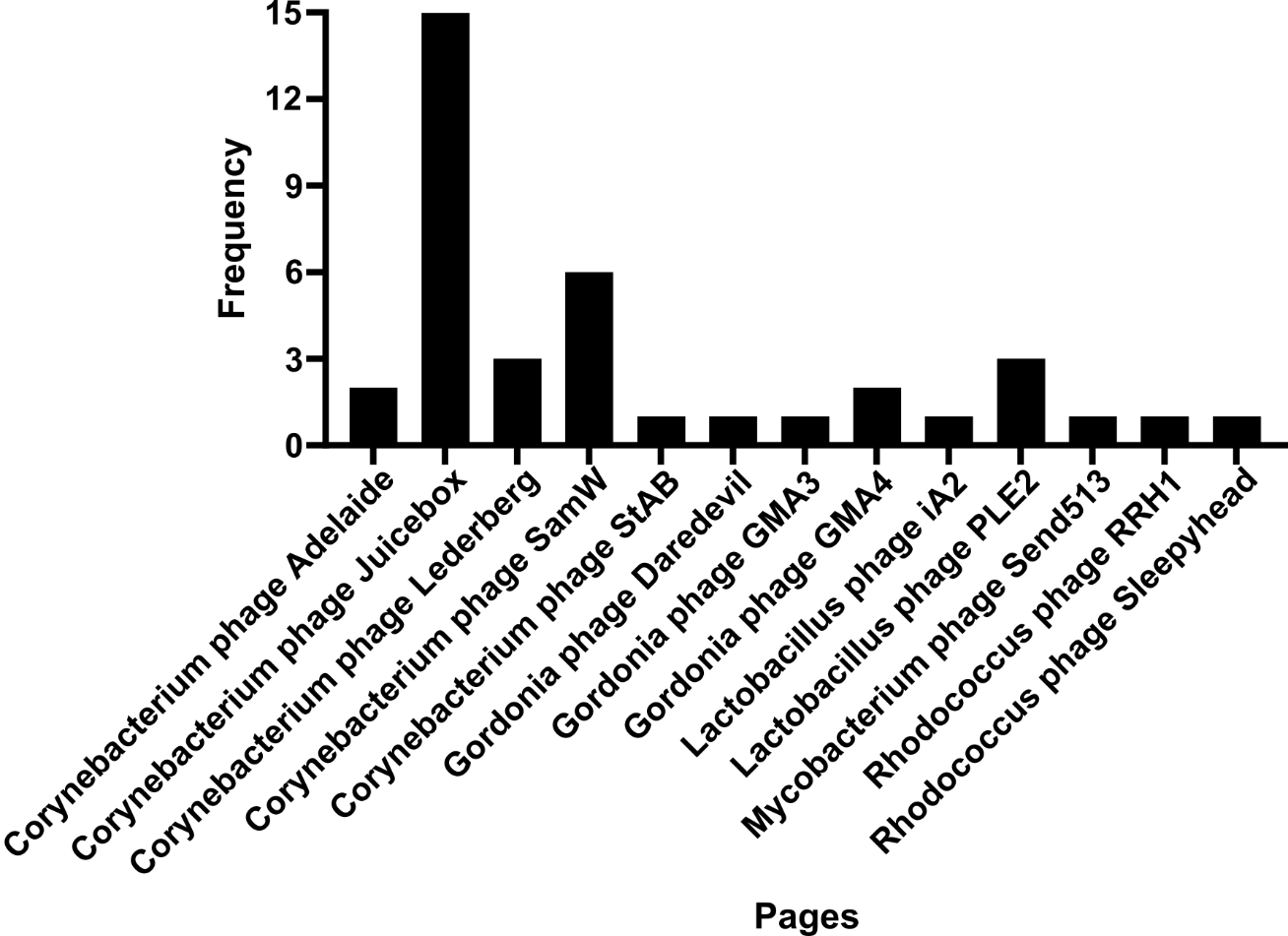
**

**Supplementary Figure S3.** Presence / absence of essential genes in mycolic acid biosynthesis.


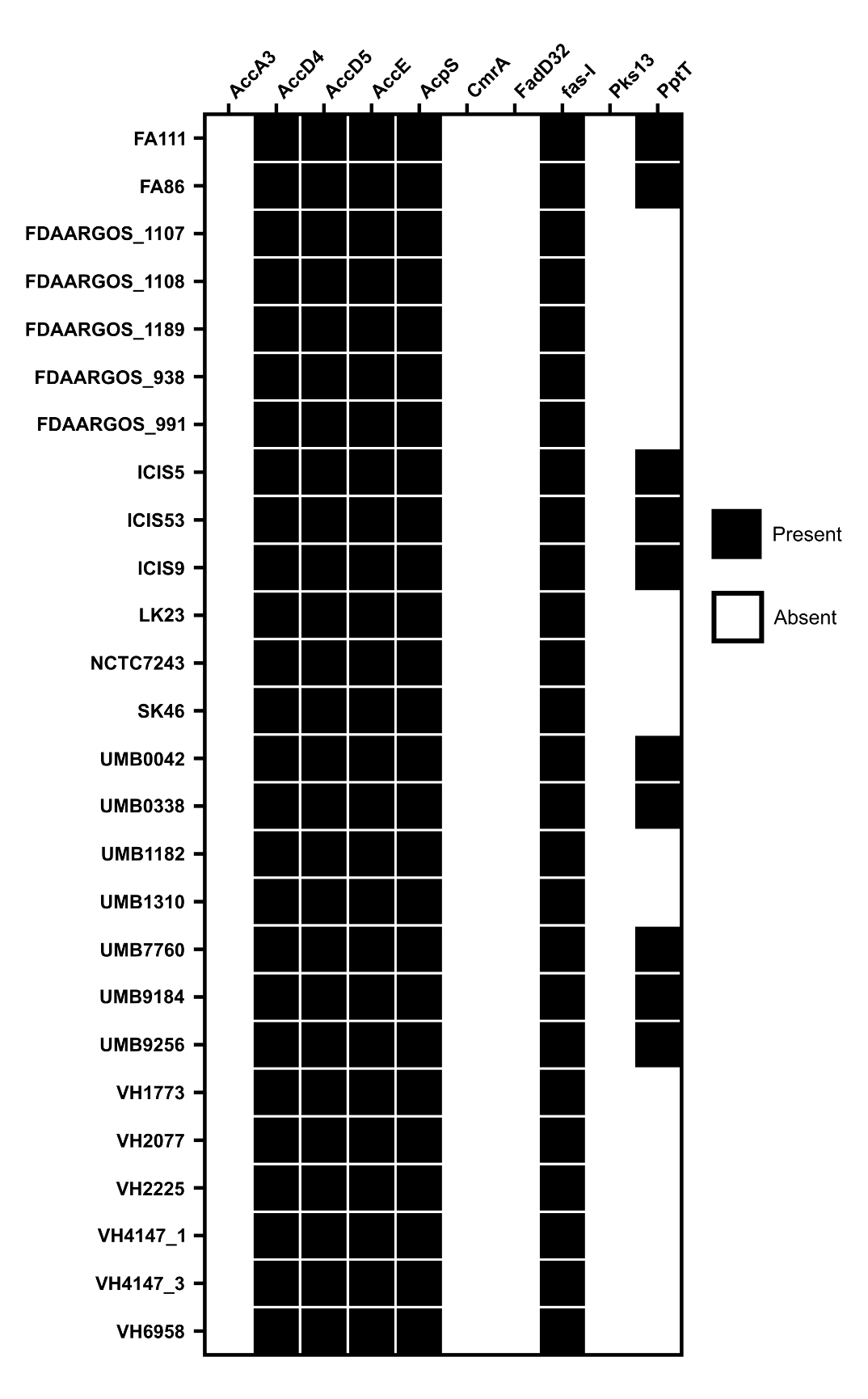

Supplement: Supplementary file 1 [file Data_Sheet_1.docx]
